# Detection and quantification of lupus anticoagulants in plasma from heparin treated patients, using addition of polybrene

**DOI:** 10.1186/1477-9560-4-3

**Published:** 2006-01-25

**Authors:** Eva M Jacobsen, Elin J Trettenes, Finn Wisløff, Ulrich Abildgaard

**Affiliations:** 1 Haematological Research Laboratory, Ullevål University Hospital, Kirkeveien 166, N-0407 Oslo, Norway; 2 Haematological Research Laboratory, Aker University Hospital, N-0514 Oslo, Norway

## Abstract

**Background:**

Lupus anticoagulants prolong clotting times in phospholipid-dependent coagulation tests. Lupus Ratio assays are integrated tests for lupus anticoagulants that may be based on APTT, RVVT or dPT clotting times. If a patient is being treated with unfractionated heparin, however, the heparin prolong clotting times and the diagnosis of lupus anticoagulant is invalidated. Commercial assays may have heparin neutralising agents added to their reagents. However, the type and efficacy of the heparin neutralisation is often not documented. We wanted to test the influence and efficacy of heparin neutralisers in the Lupus Ratio assay.

**Methods:**

Several heparin neutralisers were tested, and polybrene was chosen for further testing. Unfractionated heparin and/or polybrene were added to normal plasma and to plasma from patients with or without lupus anticoagulant and clotting times compared before and after the additions. Lupus anticoagulant-positive patients were given 5000 IU i.v. of unfractionated heparin and plasma was collected just before and five minutes after the injection. Lupus Ratios were calculated after polybrene was added to the postinjection samples.

**Results:**

The Lupus Ratio became slightly lower when polybrene was added to plasma without heparin. Plasma heparinised in vitro and plasma from patients that had received heparin, both had Lupus Ratios nearly identical to the Lupus Ratios calculated before any additions.

**Conclusion:**

By addition of polybrene to a final concentration of 7.9 μg/ml in test plasma, Lupus Ratio may be determined in lupus anticoagulant-negative as well as positive plasmas irrespective of the presence of heparin 0.0 – 1.3 U/ml.

## Background

*Lupus anticoagulants (LAs) *are a heterogeneous group of antibodies directed against phospholipidbinding (PL-binding) proteins or complexes of PLs and proteins [1]. By definition, LAs prolong clotting times in one or several PL-dependent coagulation assays (APTT, RVVT, PT) [2,3].

*The lupus ratio (LR) test *is an integrated test for LA, incorporating screening test, mixing studies, and confirmatory testing into one assay [4,5]. The LR test may be based on either one of the phospholipid-dependent clotting time assays APTT, RVVT, or dPT [4,6]. The presence of unfractionated heparin (UFH) in the sample may, however, prolong clotting times and thereby invalidate the diagnosis of LA. Schjetlein et al. tested 28 patients on treatment with UFH. 24 of these patients had false positive tests for LA in the APTT-based LR test, and 19 of the 28 patients had false positive tests in the RVVT-based LR test [4]. Liestøl et al. found that heparin concentrations in plasma exceeding 0.625 U/ml gave dPT clotting times that were immeasurably long in the dPT-based LR test [6].

In commercial assays, heparin neutralising agents are often added to the reagents to avoid this problem (LA Screen/LA Confirm, Gradipore Ltd; DVVtest/DVVconfirm, American Diagnostica Inc.; LAC Screen/LAC Confirm, Instrumentation Laboratory Ltd). However, the type of heparin neutraliser is often not specified and documentation of the efficacy of the added agents not given. There has also been some dispute on false positive test results in samples from heparinised patients even with reagents containing heparin neutralising agents [7,8].

Since heparin may be started on the suspicion of venous thromboembolism only, and these patients are discharged from hospital early and often followed by general practitioners, testing for LA is often neglected. We wanted to determine whether the inclusion of a heparin neutralising substrate in the LR assay would make it suitable for testing patients on treatment with heparin.

## Methods

### Normal plasma (NP)

Blood samples were collected from 30 presumably healthy volunteers with normal APTT. The samples were collected by venipuncture into 4.5 ml test tubes containing 0.5 ml of 0.129 M buffered sodium citrate. After centrifugation at 4000 g for 20 minutes, the plasma was pooled and stored in small aliquots at -70°C.

### Heparin neutralisers

Solutions were made of hexadimethrinebromide (Polybrene^®^, Sigma Chemical Company, Saint Louis MO, USA), poly-_L_-arginine (poly-_L_-arginine hydrochloride, MW 15,000–70,000, Sigma), poly-_L_-lysine (poly-_L_-lysine hydrobromide, MW 15,000–30,000, Sigma), poly-_DL_-lysine (poly-_DL_-lysine hydrobromide, MW 1,000–4,000, Sigma), poly-_L_-histidine (poly-_L_-histidine hydrochloride, MW 15,000–50,000, Sigma), or protamine sulphate (Protamine^® ^Sulphate, LEO, Ballerup, Denmark) and 0.15 M NaCl to concentrations of 0.66 mg/ml.

For the testing of the different heparin neutralisers, each of them was added to 0.5 ml NP to final concentrations varying from 0.0 to 6.6 μg/ml. Clotting times were measured with APTT-reagents containing crude cephalin diluted 1/100, 1/200, 1/800 or 1/3200. Clotting times with each heparin neutraliser were also measured after the addition of UFH 0.5 U/ml (final concentration) to NP.

### Lupus Ratio tests

**Figure 1:**
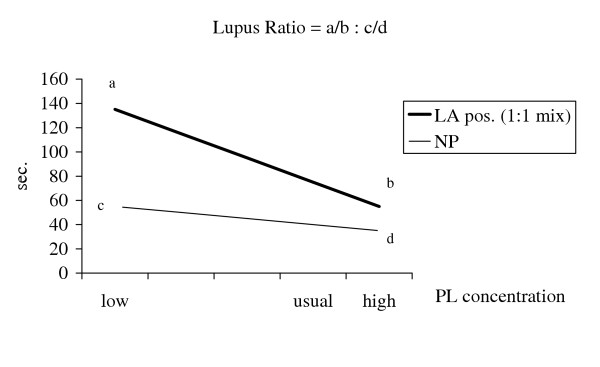
**Calculation of Lupus Ratio**. Lupus Ratio (LR) may be calculated from APTT, RVVT or dPT-based clotting times. A LR test is performed on 1:1 mixtures of patient plasma and normal plasma (NP). The clotting time is measured twice, once with a reagent with a low cephalin concentration (a), then with a reagent with a high cephalin concentration (b). The ratio between these clotting times is divided by the corresponding ratio of NP (c:d). This final ratio is the LR of that plasma (a/b : c/d).

The clotting time of 1:1 mixture of patient plasma and NP is determined with low and high PL concentrations, respectively. The ratio between these two coagulation times is normalised by dividing with the corresponding ratio of the NP. This final ratio is the Lupus Ratio (LR) of that plasma (Fig. 1).

APTT reagents were made from dilutions of crude cephalin from bovine brain (a generous gift from Axis-Shield PoC AS, Oslo, Norway). A constant concentration (final concentration 6 mg/l) of ellagic acid was used as activator. The reagents for the APTT-based Lupus Ratio test contain crude cephalin diluted 1/100 and 1/3200 in Owren's buffer, respectively [4]. In-house reagents for the RVVT-based Lupus Ratio test contain crude cephalin diluted 1/200 and 1/10000 in Owren's buffer. Russell's Viper Venom (Sigma-Aldrich Norway A/S, Oslo, Norway) was diluted in imidazole buffer with 1% BSA and aliquots were stored at -20C [4,9].

After earlier testing of stability of reagents and reproducibility of clotting times, the cephalin dilutions used in the RVVT assay are made one day ahead of their use and are stable for at least one week. The RVV was kept on ice and was stable for 4 hours after thawing. The APTT reagents were autoclaved and sealed and were then stable for months.

The reference limits have been calculated earlier from a reference population of 120 healthy blood donors [5]. The 97.5 percentile was chosen as the upper reference limit. The upper reference limit for the APTT-based assay was 1.05; for the RVVT-based assay it was 1.11.

Based on serial dilutions of plasma from patients with strong LA activity, we have previously chosen arbitrary limits for semi-quantification of LAs into weak, moderate or strong. This semi-quantification has been shown to have good reproducibility [5].

### APTT-based Lupus Ratio in test plasma heparinised in vitro

Plasma from twenty patients was collected in the same way as described for NP. The patients included twelve patients with previously diagnosed LA and eight patients with previous negative tests for LA.

The APTT-based LR test was performed on patient plasma before and after the addition of 1/50 volume of polybrene or NaCl, with a final concentration of polybrene of 7.9 μg/ml. Patient plasma with polybrene was then tested before and after the addition of 1 U/ml UFH (final concentration) or NaCl.

### RVVT-based Lupus Ratio

Polybrene with a final concentration of 3.96 μg/ml, which should be able to neutralise up to 0.6 U/ml heparin, and/or UFH 0.5 U/ml (final concentration) was added to normal plasma. RVVT-based Lupus Ratios were then measured.

Polybrene (final concentration 3.96 μg/ml) and/or UFH was also added to patient plasma from both patients with known LA (n = 12) and plasma from patients without LA (n = 13).

### Test plasma from heparinised patients

Plasma from eleven LA positive patients had been collected earlier in a study on tissue factor pathway inhibitor in LA positive patients [10]. Informed consent was obtained from all patients before inclusion. Blood samples were drawn before and exactly five minutes after the intravenous injection of 5000 IU UFH. Aliquots were stored at -70°C. The heparin concentration in postheparin plasma, measured as antiXa activity (Coatest^®^, LMW heparin/heparin, Chromogenix AB, Mölndal, Sweden), ranged from 0.50 to 1.37 U/ml (individual results not shown). Polybrene (Sigma) was added to NP to a concentration of 15.84 μg/ml. Patient plasma was mixed 1:1 with polybrene-containing NP. The LRs of these mixtures were measured.

### Coagulation instruments

The APTT of plasma heparinised in vitro was measured with the Thrombotrack instrument from NycoMed Pharma. The remaining studies were performed with STA Compact coagulation analyser from Stago Diagnostica.

## Results

### Choice of heparin neutraliser

Poly-_L_-histidine and poly-_DL_-lysine were not able to neutralise heparin and were excluded from further testing.

**Figure 2:**
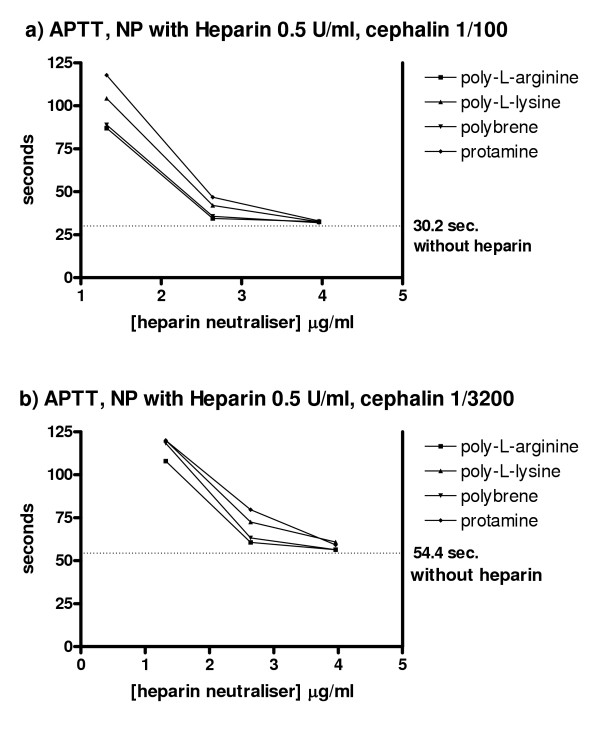
**Effect of heparin neutralisers on the APTT of heparinised plasma**. a) APTT values of Na-citrate plasma with heparin added in vitro to a final concentration of 0.5 U/ml. APTT reagent with cephalin 1/100 as phospholipid source. APTT in this heparinised plasma was unmeasurable without heparin neutraliser, i.e. clotting times >120 sec. The figure shows the clotting times when increasing concentrations of the different heparin neutralisers were added. b) APTT values of Na-citrate plasma with heparin added to a final concentration of 0.5 U/ml. APTT reagent with cephalin 1/3200 as phospholipid source. Again, APTT was >120 sec. when no heparin neutraliser were added. The figure shows the clotting times when increasing concentrations of the different heparin neutralisers were added.

The remaining four cations tested all showed a tendency to prolongation of the APTT, particularly with the APTT-reagent with low phospholipid concentration (table I). The effect of poly-_L_-arginine seemed less predictable than that of the others as it could both prolong and shorten the APTT (table I). All four cations could neutralise completely the heparin effect in the APTT assays (figure 2).

**Table 1:** Influence of heparin neutraliser on the APTT. APTT clotting times were measured in NP with two reagents with crude cephalin diluted 1/100 and 1/3200, respectively. Mean results of two experiments and percent change in clotting time with addition of any heparin neutraliser are given. All experiments were performed in triplicates.

	APTT, cephalin 1/100		APTT, cephalin 1/3200	
	
	mean, seconds	% change	mean, seconds	% change
without heparin neutraliser	30.4	--	58.7	--
Poly-L-Arginine 3.96 μg/ml	33.8	11.2	55.8	-4.94
Poly-L-Lysine 3.96 μg/ml	29.7	-2.4	79.8	36.06
Polybrene 3.96 μg/ml	34.1	12.1	72.7	23.96
Protamine 3.96 μg/ml	35.7	17.5	64.4	9.80

APTT clotting times were measured in normal plasma with two reagents with crude cephalin diluted 1/100 and 1/3200, respectively. Percent change in mean clotting times with addition of any heparin neutraliser are given.

Poly-L-arginine and polybrene were marginally more effective. As the stability of polybrene is better documented, it was chosen as heparin neutraliser.

Results obtained with a thrombin time assay indicated that 7.9 μg polybrene neutralised completely 1.2 units of UFH (results not shown). This concentration of polybrene was used in the following experiments. In the LR assay test plasma is mixed with NP prior to assay. For practical purposes, polybrene was added to the NP batch to a final concentration of 7.9 × 2 = 15.8 μg/ml, in order to obtain the same polybrene:heparin ratio as when polybrene was added directly to test plasma.

### Heparinisation in vitro

#### a) Effect on APTT and the APTT-based LR

Polybrene prolonged the APTT of NP slightly (table 1), but this prolongation was reduced or abrogated by heparin (figure 2).

Table 2 shows the results of the APTT-based LR when polybrene or polybrene and heparin was added to plasma from 20 patients. The LR was somewhat lower after polybrene was added. This affected categorisation of some plasma samples with LR values near discriminating values. Thus two patients switched from moderate to low positive, and one patient from weak positive to negative.

**Table 2:** APTT-based Lupus Ratio before and after the addition of polybrene and heparin. APTT-based Lupus Ratios were determined in LA-positive and LA-negative patient plasmas before and after the addition of polybrene 7.9 μg/ml and/or heparin 1.0 U/ml. APTT-based Lupus Ratio <1.05 = negative result.

	APTT-based Lupus Ratio median (range)	
	LA-positive patients n = 12	LA-negative patients n = 8

Lupus Ratio	1.29 (1.07 to 2.24)	0.99 (0.84 to 1.03)
Lupus ratio with polybrene	1.20 (1.01 to 2.19)	0.94 (0.82 to 1.00)
% change	-5.50 (-10.85 to +1.77)	-1.06 (-13.29 to +2.92)
Lupus ratio with heparin and polybrene	1.26 (1.04 to 2.22)	0.98 (0.85 to 1.00)
% change	-3.52 (-6.80 to --1.10)	-1.49 (-3.09 to +1.19)

The LR values obtained in plasma samples containing both polybrene and heparin were very similar to baseline values, but also here categorisation changed for three patients with LR values near discriminating values.

#### b) The effect on the RVVT and the RVVT-based LR

Polybrene had little influence on the RVVT (table 3) and the RVVT-based LR of patients with or without LA (table 4). Addition of UFH 0.5 U/ml prolonged RVVT markedly and resulted in false positive LR results (table 4). In LA positive samples, UFH prolonged the clotting times beyond 120 seconds and LR could not be calculated. In heparinised plasma, polybrene neutralised heparin and the resulting LR values were very similar to baseline LR values.

**Table 3:** Russel Viper Venom Times in normal plasma. RVVT were measured in NP with two different phospholipid reagents, with crude cephalin diluted 1/200 or 1/10 000, respectively. Addition of unfractionated heparin to plasma without polybrene gave false positive Lupus Ratios, while addition of heparin to plasma with polybrene gave correct result. Results are given as means of three runs. RVVT-based Lupus Ratio <1.11 = negative result.

Heparin concentration	Polybrene concentration	Mean clotting time with cephalin 1/200	Mean clotting time with cephalin 1/10 000	Lupus Ratio
0.0 U/ml	0.00 μg/ml	29.8 seconds	46.3 seconds	------
0.0 U/ml	3.96 μg/ml	30.4 seconds	48.8 seconds	1.03 (neg.)
0.5 U/ml	3.96 μg/ml	29.8 seconds	46.9 seconds	1.01 (neg.)
0.5 U/ml	0.00 μg/ml	38.1 seconds	75.4 seconds	1.27 (false pos.)

Russel Viper Venom Times were measured with two different reagents, with crude cephalin diluted 1/200 or 1/3200, respectively. Addition of unfractionated heparin to plasma without polybrene gave false positive Lupus Ratios, while addition of heparin to plasma with polybrene gave correct result. RVVT-based Lupus Ratio ≤ 1.10 = negative.

**Table 4:** RVVT-based Lupus Ratio before and after the addition of polybrene and heparin. RVVT-based Lupus Ratios were determined in LA-positive and LA-negative patient plasmas before and after the addition of polybrene 3.96 μg/ml and/or heparin 0.5 U/ml. RVVT-based Lupus Ratio <1.11 = negative result.

	RVVT-based Lupus Ratio median (range)	
	LA-positive patients n = 12	LA-negative patients n = 13

Lupus Ratio	1.33 (1.14 to 1.63)	0.99 (0.88 to 1.06)
Lupus ratio with polybrene	1.36 (1.20 to 1.69)	1.03 (0.92 to 1.11)
% change	2.33 (-1.52 to +10.16)	2.94 (-1.02 to +9.57)
Lupus ratio with heparin and polybrene	1.32 (1.16 to 1.52)	1.01 (0.87 to 1.08)
% change	-2.51 (-9.40 to +6.78)	1.09 (-3.03 to +5.21)

### Heparin treated patients

The median preheparin APTT-based LR in the eleven patients that received one injection with UFH was 1.40 (table 5). LR values with polybrene, both in heparinised and preheparin samples, were very similar to baseline LR values (median LR with polybrene 1.34; median LR in postheparin samples 1.32). In one patient the semi-quantification changed from moderate to low positive after the injection of heparin (table 5).

**Table 5:** APTT-based Lupus Ratio in heparinised patients. Blood samples were collected from 11 patients with previous positive tests for LA before and five minutes after i.v. injection of unfractionated heparin 5000 IU. Polybrene was added to plasma with a final concentration of 7.9 μg/ml. (+) = weak positive LR; (++) = moderate positive LR; (+++) = strong positive LR.

Patient	Anti-Xa activity after heparin i.v.	LR before heparin	LR before heparin, polybrene added	Change %	LR after heparin i.v. polybrene added	Change %
1	1.26	1.79 (+++)	1.87 (+++)	4.47	1.72 (+++)	-3.91
2	0.50	1.21 (+)	1.23 (+)	1.65	1.22 (+)	0.83
3	0.65	1.76 (+++)	1.79 (+++)	1.70	1.81 (+++)	2.84
4	1.12	2.02 (+++)	1.96 (+++)	-2.97	1.92 (+++)	-4.95
5	1.15	1.05 (+)	1.05 (+)	0.00	1.08 (+)	2.86
6	1.20	1.40 (++)	1.32 (++)	-5.71	1.32 (++)	-5.71
7	1.23	1.15 (+)	1.17 (+)	1.74	1.19 (+)	3.48
8	1.11	1.54 (+++)	1.64 (+++)	6.49	1.66 (+++)	7.79
9	1.37	1.30 (++)	1.34 (++)	3.08	1.21 (+)	-6.92
10	0.72	1.33 (++)	1.34 (++)	0.75	1.31 (++)	-1.50
11	1.05	2.02 (+++)	1.92 (+++)	-4.95	1.85 (+++)	-8.42

Median		1.40	1.34	1.65	1.32	-1.50
Range		1.05 to 2.02	1.05 to 1.96	-5.71 to +6.49	1.08 to 1.92	-8.42 to +7.79

## Discussion

Various polyamino acids, protamine, and polybrene are all polycations that may neutralise the heparin effect in clotting assays [11,12]. The polycations neutralise the negatively charged heparin by forming inactive complexes [13]. Protamine, derived from the sperm of salmon, was the first heparin neutraliser to be described [14]. Some of the synthetic polyamino acids may show a wider range of neutralisation than protamine [15]. Polybrene is a synthetic polymerised quaternary ammonium salt [16] and was reported to be more stable in plasma than protamine [11]. The chosen concentration of polybrene (7.9 μg/ml) was able to neutralise heparin 1.2 U/ml completely. The principle of LR has proved to give robust tests with reproducible results [4-6]. When the APTT-based clotting time results of an international interlaboratory study were used to calculate the results according to different principles used in LA testing, the calculation of a LR proved to give the highest sensitivity and high concordance between the laboratories [17]. However, heparinised plasma may give false positive results [4,6]. The results in this report show that the Lupus Ratio can be reliably calculated in heparinised plasma after neutralisation with polybrene. Polybrene prolonged the APTT somewhat, but influenced the LR very little. Only one negative test result became positive after the addition of polybrene, and one positive test result became negative. In both cases the positive results were borderline values. There were very few changes in the categorisation of the positive test results.

Our results show that one may add polybrene to all the NP used in the LR assays. In this way, one does not have to use a different procedure for plasma containing UFH compared to samples from patients that have not received UFH.

Our LR assays are in-house tests. The reagents used in different in-house and commercial APTT and RVVT-based assays for LA differ in their composition of phospholipids. The amount and type of contact activator in the APTT-based tests are not standardised. Therefore, the results here may not automatically be assigned to other test systems.

Testing for lupus anticoagulant is an important part of the evaluation of patients with venous or arterial thrombosis. Positive test results should, however, always be confirmed with repeated testing at least 4–6 weeks after the initial positive finding. Repeated positive findings of antiphospholipid antibodies in a patient that has experienced a thrombotic episode indicates an increased risk of recurrence [18,19], and prolonged treatment may be indicated. Often these patients have not been tested before treatment is started. Since these patients are discharged early and subsequent care may be decentralized, postponing the testing can mean neglected testing. Testing for LA in heparinised plasma after neutralisation with polybrene may ensure that the patient gets a complete evaluation of thrombophilia factors.

## Conclusion

Unfractionated heparin prolong clotting times and testing for LA should preferably be performed in plasma without heparin. However, in this paper we have showed that by addition of polybrene to a final concentration of 7.9 μg/ml in test plasma, LR may be determined in LA negative as well as positive plasmas irrespective of the presence of heparin 0.0 – 1.3 U/ml.

## Competing interests

The author(s) declare that they have no competing interests.

## Authors' contributions

EMJ recruited patients and performed the heparin injection experiment. She also drafted the manuscript. EJT performed the clotting assays. FW participated in the design of the study and contributed to the drafting of the manuscript. UA had main responsibility for the design of the study and contributed to the drafting of the manuscript. All authors read and approved the final manuscript.
